# Lysophosphatidic acid improves corneal endothelial density in tissue culture by stimulating stromal secretion of interleukin‐1β

**DOI:** 10.1111/jcmm.15307

**Published:** 2020-04-24

**Authors:** Yi‐Jen Hsueh, Yaa‐Jyuhn James Meir, Jui‐Yang Lai, Hung‐Chi Chen, David Hui‐Kang Ma, Chieh‐Cheng Huang, Tsai‐Te Lu, Chao‐Min Cheng, Wei‐Chi Wu

**Affiliations:** ^1^ Department of Ophthalmology Chang Gung Memorial Hospital Linkou Taiwan; ^2^ Center for Tissue Engineering Chang Gung Memorial Hospital Linkou Taiwan; ^3^ Department of Biomedical Sciences Chang Gung University Taoyuan Taiwan; ^4^ Institute of Biochemical and Biomedical Engineering Chang Gung University Taoyuan Taiwan; ^5^ Department of Materials Engineering Ming Chi University of Technology New Taipei City Taiwan; ^6^ Department of Medicine Chang Gung University Taoyuan Taiwan; ^7^ Department of Chinese Medicine Chang Gung University Taoyuan Taiwan; ^8^ Institute of Biomedical Engineering National Tsing Hua University Hsinchu Taiwan

**Keywords:** corneal endothelial cell (CEC), Interleukin‐1β (IL‐1β), lysophosphatidic acid (LPA), stromal‐endothelial interactions, tissue culture

## Abstract

The short supply of donor corneas is exacerbated by the unsuitability of donors with insufficient endothelial cell density. Few studies have investigated promoting corneal endothelial cell proliferation to increase the endothelial cell density. We hypothesize that pre‐transplantation treatment of proliferative tissue‐cultivated corneas may increase corneal endothelial cell density. We observed that the airlift cultures were superior to immersion cultures with respect to both transparency and thickness. In this tissue culture system, we observed that lysophosphatidic acid increased the rabbit corneal endothelial cell density, number of BrdU‐positive cells and improve wound healing. We also observed an indirect effect of lysophosphatidic acid on corneal endothelial cell proliferation mediated by the stimulation of interleukin‐1β secretion from stromal cells. Human corneal tissues treated with lysophosphatidic acid or interleukin‐1β contained significantly more Ki‐67‐positive cells than untreated group. The lysophosphatidic acid‐ or interleukin‐1β‐treated cultured tissue remained hexagon‐shaped, with ZO‐1 expression and no evidence of the endothelial‐mesenchymal transition. Our novel protocol of tissue culture may be applicable for eye banks to optimize corneal grafting.

## 1 INTRODUCTION

The corneal stroma confers a transparent property that allows the transmission of light into the globe. This transparency is affected by water content, which is regulated by a monolayer of corneal endothelial cells (CECs) that actively pump water out of the corneal stroma into the anterior chamber. Clinically, only donor corneas with an endothelial cell density (ECD) greater than 2100 cells/mm^2^ are eligible for transplantation, because the ECD is a critical indicator for post‐operative follow‐up and assessments.^1^ This requirement diminishes the supply of corneas suitable for transplantation. Increasing the ECD in otherwise unsuitable corneas is technically challenging, as the mature corneal endothelial monolayer remains in a non‐proliferative state, and human CECs are generally considered not expandable in vivo and only slowly expandable in vitro.^2^

The storage of donor corneas after harvesting presents a number of potential hazards to CECs, whether stored as corneoscleral buttons in Optisol GS medium at 4°C^3^ or as whole globes in CorneaPrep/Max medium at 31°C.^4^ The hypothermic preservation of corneoscleral buttons is rather popular at eye banks in many countries, including the US and Taiwan, because these corneas are less oedematous and to easier access during surgery. However, the significant correlation between storage time and a decrease in ECD is a great disadvantage of this method.^5^ To address this problem, we seek to identify pertinent pre‐clinical treatments for proliferative tissue‐cultured corneas that can revive declining CECs and increase the ECD, thereby increasing the availability of suitable donor corneas.

With rapid advancements in corneal endothelial biology, it is not only possible to cultivate CECs in vitro but also feasible to develop tissue engineering strategies for the fabrication of transplantable CEC sheets.^6^, ^7^ Treatments reported to induce proliferation in post‐mitotic CECs include EDTA^8^ and a Rho‐associated protein kinase (ROCK) inhibitor.^9^ Previously, we observed functional p120‐Kaiso signalling in both retinal pigment epithelium and CECs and showed that the down‐regulation of p120 resulted in proliferation without disrupting cell junctions.^10^, ^11^ Additionally, significant expression of yes‐associated protein 1 (YAP), a Hippo pathway core protein, was observed in the cell nuclei.^11^ We also demonstrated that treatment with a natural compound, lysophosphatidic acid (LPA), increased nuclear YAP, triggering cell cycle progression in the contact‐inhibited monolayer of human CECs.^12^ Here we aim to investigate whether LPA treatment of tissue‐cultured donor corneas before transplantation increases CEC proliferation.

Stromal‐epithelial interactions regulate the proliferation and differentiation of corneal epithelial cells via stroma‐derived paracrine secretion.^13^, ^14^, ^15^, ^16^, ^17^ Upon corneal injury, corneal stromal cells (CSCs), also known as ‘keratocytes’, transform into active fibroblasts in which the expression of keratocan (a keratocyte marker) is down‐regulated.^18^ Active fibroblasts secrete factors such as hepatocyte growth factor (HGF) to stimulate the proliferation and migration of epithelial cells.^19^ CSC‐derived secretion has been evidenced to facilitate corneal endothelial wound healing^20^ and to inhibit endothelial‐mesenchymal transition through secretion of HGF.^21^ However, the existence and mechanisms of interactions between stromal and endothelial cells remain unclear.

Conventionally, corneal donor culture research predominantly focused on the preservation of donor corneas.^22^, ^23^, ^24^, ^25^ Few studies focused on promoting the proliferation of CECs.^26^ Here, we established an airlift corneal tissue culture system in which the ECD increased significantly upon addition of LPA to the medium. We also observed that stroma‐derived interleukin‐1β secretion was involved in LPA‐induced cell proliferation in this tissue culture system.

## 2 MATERIALS AND METHODS

### 2.1 Materials

DMEM, Opti‐MEM, human endothelium serum‐free medium (HESFM), gentamicin, amphotericin B, trypsin‐EDTA, FBS, PBS and an Alexa Fluor‐conjugated secondary IgG antibody were purchased from Invitrogen. Collagenase A was purchased from Roche Applied Science. Alizarin Red, RPMI 1640 vitamin solution, lysophosphatidic acid (LPA), mitomycin C, Hoechst 33342 dye, paraformaldehyde and Triton X‐100 were purchased from Sigma‐Aldrich. Recombinant human proteins of fibroblast growth factor‐basic (b‐FGF), interleukin‐1β (IL‐1B) and chemokine (C‐C motif) ligand 20 (CCL20) were purchased from Peprotech. Recombinant human epidermal growth factor (hEGF) was purchased from Upstate, Millipore. FNC coating mix (FNC) was purchased from Athena ES. All plastic cell culture dish was obtained from Corning Incorporated Life Sciences.

The following antibodies were purchased: mouse anti‐ZO‐1 antibody (clone ZO1‐1A12), Invitrogen; mouse anti‐BrdU antibody (RPN20Ab), Amersham GE Healthcare; mouse anti‐interleukin‐1β antibody (clone AS20), MyBioSource; rabbit anti‐Ki‐67 antibody (H‐300), Santa Cruz Biotechnology; and rabbit anti‐keratocan antibody (ab113115), Abcam.

### 2.2 Sources of human and animal corneas

The tenets of the Declaration of Helsinki were followed in the current study, which was approved by the Institutional Review Board of Chang Gung Memorial Hospital (No. 104‐9358B). The need for consent from the donors was waived because the human tissues used were delinked and discarded after surgery. Within 7 days after PK at Chang Gung Memorial Hospital, the residual human corneoscleral tissues were maintained at 4°C in Optisol (Chiron Vision). In total, 10 corneas from donors were subjected to examinations; of these, four were used to assess the TC medium, and six were used for tissue culture.

Corneal tissues were obtained from 4‐month‐old New Zealand white rabbits immediately after euthanization, and the tissues were stored in 50‐mL tubes containing DMEM for further examinations. All animals were raised in the Animal Care Core Facility of Chang Gung Memorial Hospital, Linkou. The protocol for the use of rabbit corneas was in accordance with the Association for Research in Vision and Ophthalmology Statement for the Use of Animals in Ophthalmic and Vision Research.

### 2.3 Cell preparation

The procedures for the isolation and culture of human and rabbit CECs were modified from previous methods.^12^ Briefly, the Descemet's membrane (DM containing CECs) was stripped from the posterior surface of corneal tissues. The DM fragments were removed and digested at 37°C for 16 hours with 2 mg/mL collagenase A in Opti‐MEM containing 50 μg/mL gentamicin and 5 μg/mL amphotericin B. After digestion, the CEC aggregates were collected by centrifugation and then cultured on 24‐well plates (Nalgene Nunc International) coated with FNC. The human CEC aggregates were maintained in HCEC medium (Opti‐MEM supplemented with 10% FBS, 20 ng/mL hEGF, 10 ng/mL b‐FGF, RPMI 1640 vitamin solution [Sigma‐Aldrich], 25 μg/mL gentamicin and 1.25 μg/mL amphotericin B). The rabbit CEC aggregates were maintained in RCEC medium (DMEM supplemented with 10% FBS and 25 μg/mL gentamicin) and subcultured for future investigations. Immortalized HCECs (B4G12) were purchased from Creative Bioarray (NY) and cultured in B4G12 medium (HESFM supplemented with 2% FBS and 10 ng/mL b‐FGF.

To isolate rabbit corneal stromal cells (RCSCs), central buttons were punched out of DM‐stripped corneas using a Barron Donor Cornea Punch (Katena) and incubated in 5 mg/mL dispase II at 4°C overnight. Loose epithelial sheets were removed, and stromal discs were cut into small pieces and digested using 2 mg/mL collagenase I for 24 hours at 37°C. The cell pellets were collected via centrifugation (1000 rpm, 25°C, 5 minutes). The RCSCs were resuspended in RCSC medium (DMEM supplemented with 10% FBS and 25 μg/mL gentamicin) in 24‐well plates coated with FNC and subcultured for further investigation. Primary human CSCs (HCSCs) (Innoprot) were cultured in HCSC medium (DMEM supplemented with 10% FBS, ITS, 1 μg/mL amphotericin, and 25 μg/mL gentamicin). All cells were cultivated and incubated at 37°C with 5% CO_2_.

### 2.4 Establishment of an airlift tissue culture system

For human corneal tissue culture, sclerocorneal residual tissues were placed endothelial side up in culture dishes and incubated with HCEC or TC medium (composed of DMEM supplemented with 10% FBS and 25 μg/mL gentamicin) at 37°C. Rabbit corneal tissues were cultivated either under immersion or in an airlift system using TC medium. To establish the airlift tissue culture system, a 1‐mL pipette tip (8 mm diameter) was cut 10 mm from the tip and fixed with n‐butyl‐2‐cyanoacrylate glue (Histoacryl; B. Braun) to the centre of a 12‐well dish. Rabbit corneal tissues were placed epithelial side up on the hollow column, partly immersed in the TC medium, keeping the epithelial surface in contact with the air. The corneal stromal thickness was measured with a mini digital thickness gauge (Shahe). All tissues were incubated at 37°C in 5% CO_2_.

### 2.5 Examination of cell morphology and ECD in tissue‐cultured corneas

The corneal endothelium was observed using a phase contrast microscope, immunostained with ZO‐1, or stained with Alizarin Red to visualize cell borders of the entire endothelial mosaic. Before staining, the endothelial side was rinsed with PBS. The dye (0.5% Alizarin Red dissolved in 0.9% sodium chloride, pH adjusted to 5.2) was filtered through a 0.2‐μm filter and placed in the corneal concavity for 2 minutes. After removing the excess dye, the corneas were washed for 2 minutes in PBS and photographed under an inverted microscope. Corneal ECD was assessed by counting cells in randomly selected areas measuring 100 × 100 mm (n = 6) (Figures 2 and 5) and 200 × 200 mm (n = 3) (Figure 2). Cell shape (including aspect ratio and circularity) was quantitatively examined using ImageJ software (n = 10).

### 2.6 Assessment of cell proliferation

In the ex vivo cultured corneal endothelium, cell proliferation was examined using Ki‐67 immunostaining for human tissues or a cell proliferation kit (GE Healthcare, BrdU labelling‐based method) for rabbit tissues according to the manufacturer's instructions. Briefly, tissue‐cultured corneas were treated with or without LPA. After 2 days, BrdU was added at a final concentration of 50 μmol/L in the medium for 24 hours. The incorporated BrdU was detected using a BrdU antibody and Alexa Fluor‐conjugated secondary antibody.

In the in vitro cultured CECs, overall cell yield was assessed by cell counting for indirect assessments of differences in proliferation rate. Briefly, cells from the different groups were trypsinized and the cell suspension mixed with trypan blue. The mixture was loaded onto a haemocytometer and examined under an inverted microscope at 100 × magnification. Cell counts were averaged and then multiplied by the dilution factor. The counting was repeated 3 times for each group.

### 2.7 Transplantation of tissue‐cultured corneas into rabbits

The water homeostasis function of the corneal endothelium in tissue‐cultured corneas was evaluated in a New Zealand rabbit animal model of PK. The rabbits were anesthetized, and the conjunctival cul‐de‐sac was sterilized by irrigation with 1:1 diluted beta‐iodine. A 7.0 mm corneal button was removed from the rabbit's eye using a Hessburg‐Barron vacuum trephine (Katena). Corneal buttons (7.5 mm) were prepared from tissue‐cultured samples using a Barron Donor Cornea Punch (Katena) and sutured into place in recipient rabbits. Four cardinal sutures of 10‐0 nylon were placed, followed by at least 12 interrupted 10‐0 nylon sutures. After surgery, all animals received topical prednisolone acetate ophthalmic suspension 1% (Pred Forte; Allergan, Inc.) and 0.5% levofloxacin hydrate ophthalmic solution (Cravit; Santen Pharmaceutical), both with QID dosing for 2‐4 weeks.

### 2.8 Establishing a damage model of the corneal endothelium

To establish evenly distributed wounded regions of similar sizes, we designed a polyvinylidene difluoride (PVDF) filter‐damage method. Matrix holes were made in PVDF membranes using a needle (18G, 1.25 mm diameter). The resulting PVDF filter had a drilled surface coverage of approximately 50.5%. Following sterilization with 75% alcohol, the PVDF filter was immersed in TC medium for use.

To fabricate a damaged corneal endothelium, rabbit corneas were placed endothelial side up, covered with the PVDF filter and immersed in TC medium. The regions to be wounded were visualized under a surgical microscope, and the CECs were carefully scraped using Cellulose Eye Spears (Surgistar Inc.) and washed with TC medium. The damage was evaluated using Alizarin Red S staining, which deeply stains acellular areas, indicating the denuded DM (Figure 2D). For tissue culture and corneal transplantation, corneal damage was created using the same procedure and evaluated by phase contrast microscopy.

### 2.9 Flat‐mount immunofluorescence staining

Tissue‐cultured corneas were fixed in 4% paraformaldehyde for 15 minutes at room temperature, rinsed with PBS, permeabilized with 0.2% Triton X‐100 for 15 minutes and then rinsed again with PBS. To block non‐specific staining, the cells were incubated with 2% BSA for 30 minutes before incubation with primary antibodies (all at 1:100 dilution) for 24 hours at 4°C. After washing 3 times with PBS, the sections were incubated at room temperature for 1 hour with the corresponding Alexa Fluor‐conjugated secondary IgG and then counterstained with Hoechst 33342. Specimens were mounted with Gel Mount (Biomeda) and examined under a Zeiss fluorescence microscope or a confocal microscope (Leica). Each staining was repeated three times.

### 2.10 Protein extraction and western blotting

Total tissue lysates were prepared in Tissue Protein Extraction Reagent (T‐PER; Pierce) supplemented with 10 mmol/L sodium fluoride, 10 mmol/L sodium orthovanadate, and 1 × protease inhibitor cocktail (Sigma‐Aldrich). After carefully cutting the samples, tiny corneal stroma pieces were transferred to a microfuge tube on ice, sonicated and centrifuged for 15 minutes at 4°C at maximum speed. The supernatants were collected. Protein concentrations were determined using a Bio‐Rad protein assay kit (Bio‐Rad).

The protein extracts were resolved on 10% acrylamide gels and transferred to polyvinylidene difluoride (PVDF) membranes (Millipore), which were then blocked with 5% (w/v) fat‐free milk in PBST (PBS containing 0.05% [v/v] Tween‐20) and probed with the desired primary antibodies at a 1:1000 dilution for all antibodies except anti‐β‐actin (1:10 000 dilution) by incubation at 4°C overnight, followed by incubation with appropriate horseradish peroxidase‐conjugated secondary antibodies. The immunoreactive protein bands were visualized via increased chemiluminescence (ECL; GE Healthcare).

### 2.11 Gene expression analysis of secreted protein using cDNA microarray

Gene expression profiles were analysed using an Affymetrix GeneChip human Clariom D array (PN 902923, Life Technologies). Briefly, total RNA from HCSCs after 2 days of culture in TC medium (with or without 20 μmol/L LPA) was isolated with the TRIzol Reagent (Invitrogen) according to the manufacturer's instructions. Isolated RNA was then purified using an RNeasy column, followed by quality monitoring using a Bioanalyzer (Agilent Technologies). Total RNA amplification, labelling and processing were performed according to the instructions in the Affymetrix user manual for the GeneChip WT PLUS Reagent Kit. After cDNA labelling and GeneChip hybridization, a GeneArray G7 scanner (Affymetrix) was used to scan the results, which were analysed using Microarray Suite Version 5 software (Affymetrix) and converted to gene expression levels (EScores) using the Probe Profiler software (Corimbia). The cytokine genes were further screened using DAVID.

### 2.12 Antibody‐based cytokine array

The HCSC CM was treated with or without 20 μmol/L LPA for 2 days, and cytokine expression was analysed using a human cytokine antibody array (RAYBIO Antibody array G series‐5 [AAH‐CYT‐G5], RayBiotech) according to the manufacturer's instructions. The CM from each group was loaded onto a glass slide for cytokine profiling. Glass slides were placed in the Axon GenePix 4000B scanner (Axon Instruments) for measurement at an excitation frequency of 532 nm. The results were retrieved using Axon GenePix Pro 6.1.0.4 software. The median signal intensity of each target cytokine was presented as a fold change relative to that of an internal positive control.

### 2.13 Enzyme‐linked immunosorbent assays

The B4G12 cells were treated with 20 μmol/L LPA for 2 days, and cell culture supernatants were collected. The protein levels of CCL20 and IL‐1β in the supernatant were determined using a solid‐phase sandwich Enzyme‐linked immunosorbent assays (ELISA) kit (EK0453 and EK0391, Boster Immunoleader) according to the manufacturer's protocol. Absorbance was measured at 450 nm, and concentrations (pg/mL) were calculated using a 7‐point standard curve.

### 2.14 Statistical analysis

All numeric data are reported as the mean ± standard deviation. The data were compared using Student's unpaired *t* test with Microsoft Excel version 2016 (Microsoft) and analysed using 2‐tailed *P*‐values, where *P* < .05* and *P* < .01** were considered significant.

## 3 RESULTS

### 3.1 Establishment of a corneal tissue culture system

**Figure 1:**
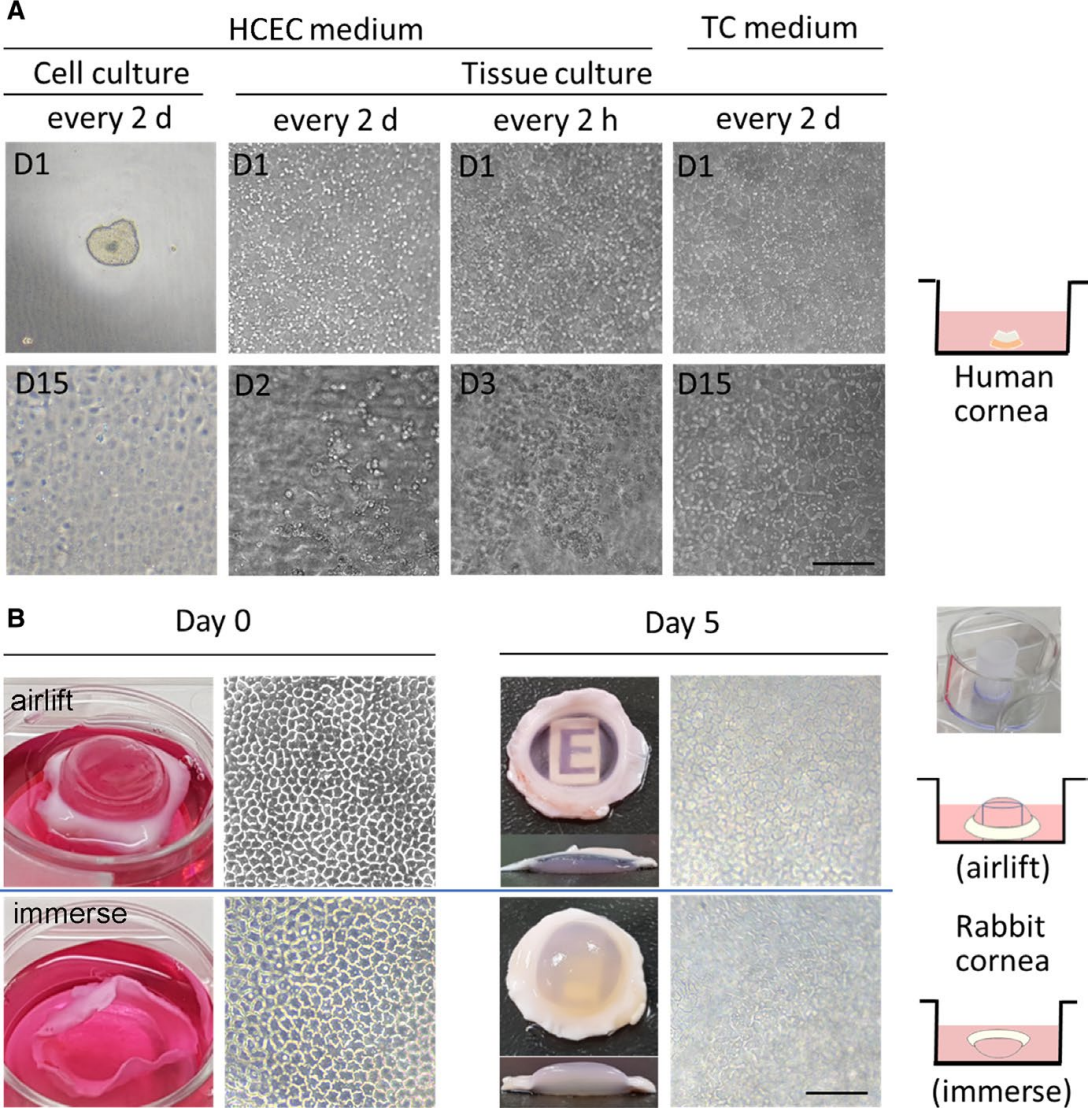
Establishment of corneal tissue culture system. A, The medium requirement for ex vivo and in vitro tissue cultures differs. To compare the culture conditions and their requirements, we adopted the following combination of media and protocols. For in vitro cell culture, the human corneal endothelium was stripped and digested by collagenase and then maintained in HCEC medium at 37°C, which was changed once every 2 d. For ex vivo tissue culture, human corneoscleral residual tissue was placed endothelial side up in the HCEC medium or tissue culture (TC) medium, which was changed once every 2 h or 2 d. The morphology of the corneal endothelium was observed using phase contrast microscopy. B, Alleviation of corneal oedema by airlift in tissue culture. Upper panel (airlift culture): After cutting off the tip of a 1‐mL pipette, the remaining hollow column was fixed to the centre of a 12‐well dish with BioGlue. Rabbit corneal tissues then were placed epithelial side up on the hollow column in TC medium, keeping the epithelial side in contact with the air. Lower panel (immersion culture): Rabbit corneal tissues were placed endothelial side up in TC medium. The gross appearance, transparency, and morphology of the corneal endothelium were observed. Scale bars represent 100 μm

For in vitro human CEC (HCEC) culture, we designed a growth factor‐supplemented medium (HCEC medium)^12^ based on previous studies.^8^, ^27^, ^28^ In HCEC medium, isolated HCECs remained proliferative for several weeks. However, when human corneal tissues were cultivated in HCEC medium, HCECs became rounded in shape and sloughed off within 48 hours (Figure 1A, left panel). Given that the environments of ex vivo tissue culture are more complicated than those of in vitro cell culture, modification of culture conditions to maintain cell viability is mandatory. The HCEC medium in the corneal tissue ex vivo culture became acidified much faster than in in vitro HCEC cultures, indicating that marked metabolic activity occurred in the donor cornea. Therefore, we replaced the medium every 2 hours and observed improved HCEC attachments, but the cell morphology remained aberrant; the cells began to detach 3 days later (Figure 1A, middle panel). Finally, we used a formula of tissue culture medium (TC medium) modified from the M5 medium by Peh et al.^29^, ^30^, ^31^ The cell shape of CECs remained normal for at least 2 weeks in this medium (Figure 1A, right panel).

We chose rabbit rather than human corneal tissue culture for subsequent studies based on the following facts. First, the availability of research corneas with an intact corneal endothelium is very limited. Second, the use of residual donor corneal specimens is not ideal, as the boundary where the central button is punched out leaves the stromal cross‐sections exposed to the culture medium, which disrupts water homeostasis in the tissue. Third, the use of rabbit corneas allows experimentation in an animal model, as immune rejection could be an issue if human corneas are implanted into rabbit eyes.

We observed that rabbit corneas under immersion culture were significantly thickened and lost their transparency, with a vague phase contrast image in the endothelium layer (Figure 1B, lower panel). Therefore, we designed an airlift tissue culture system to mimic the physiological environment in the anterior chamber. As shown in Figure 1B (upper panel), the airlifted culture was superior to the immersion culture with respect to both transparency and thickness (1281.5 μm ± 110.6 μm vs 3421.2 μm ± 159 μm, *P* < .01), with a rather clear phase contrast image in the corneal endothelium.

### 3.2 LPA treatment increases rabbit corneal endothelial cell density, CEC proliferation and wound healing in tissue culture

**Figure 2:**
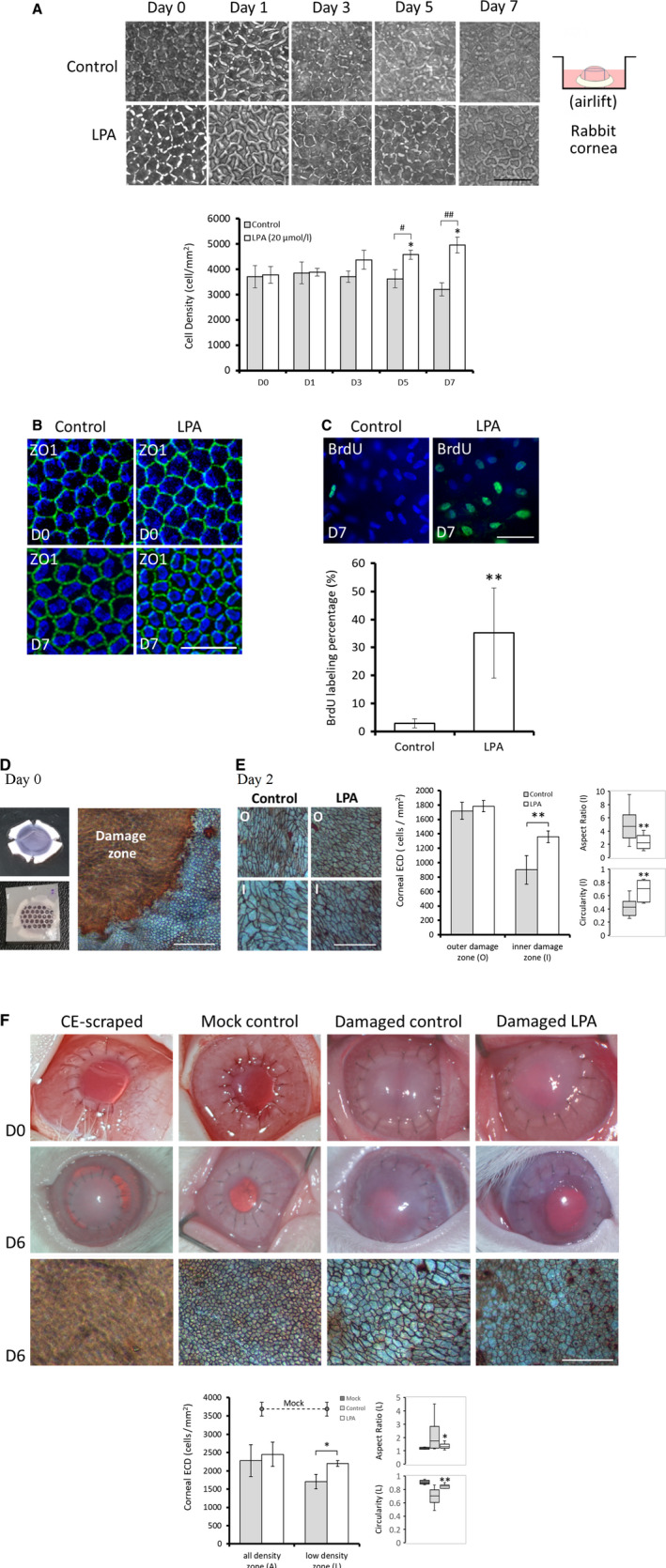
LPA increases rabbit corneal endothelial cell density (ECD), endothelial cell (CEC) proliferation, and wound healing i`n a tissue culture system. A, Rabbit corneal tissues were cultivated in an airlift tissue culture system. Phase contrast images were obtained on Days 0, 1, 3, 5 and 7. The ECD of the corneal endothelium was significantly higher in the 20 μmol/L LPA‐treated group on Days 5 and 7 (n = 3; *P < .5, vs LPA group (D 0); ^#^ *P* < .05, ^##^ *P* < .01 vs control group). B, Immunostaining of the tissue‐cultured corneal endothelium with ZO‐1 (green) revealed a normal hexagonal phenotype in both groups on Days 0 and 7. C, Cell proliferation was examined via BrdU labelling assay. After adding BrdU to the tissue culture from Days 5‐7, the number of BrdU‐labelled cells (green) was significantly higher in the LPA‐treated group (n = 3; *P < .05). The nuclei were counterstained with Hoechst 33342. Scale bars represent 50 μm. D‐E, To evaluate the healing ability of rabbit corneal endothelial cells cultured with LPA, the corneal endothelium was damaged using PVDF filters (see Section 2). Damaged corneas were airlift‐cultured in medium with or without LPA to Day 0 or Day 2 and stained with Alizarin Red S to delineate the corneal endothelium. The morphology of the corneal endothelium in the outer damage zone (O) and inner damage zone (I) are shown. In the inner damage zone, the corneal ECD and circularity were significantly greater and the aspect ratio significantly lower in the LPA‐treated group than in the control group. F, Tissue‐cultured donor corneas cultured in medium with or without LPA for 2 d were transplanted into rabbit eyes. Donor controls included corneas with total endothelium scraping and those with no damage. External eye photographs were taken on Days 0 and 6. Following sacrifice on Day 6, the corneal endothelium was stained with Alizarin Red S. In the low‐density zone (L), the average ECD and circularity were significantly higher and the aspect ratio significantly lower in those treated with LPA than in those without. Scale bars represent 200 μm. (n = 3; *P < .05, **P < .01)

ROCK inhibitor (Y27632) and p120‐catenin siRNA (si‐p120) are reported to increase the proliferation of in vitro HCECs.^9^, ^11^, ^32^, ^33^ However, in the airlift tissue culture system, treatment with Y27632 (2 μmol/L) or si‐p120 (100 nmol/L) failed to increase the ECD of rabbit corneas (Figure S1). We previously reported that LPA (20 μmol/L) increases the proliferation of in vitro‐cultivated HCECs.^12^ Therefore, LPA was added to the TC medium. As shown in Figure 2A, the central ECD of LPA‐treated rabbit corneas increased with time and was significantly higher on Days 5 and 7 as compared to Day 0 (D0 of 3766.7 ± 332.9; D5 of 4572.2 ± 179.8, *P* < .05; D7 of 4950.0 ± 304.1, *P* < .05). In contrast, the central ECD of rabbit corneas in the control group remained unchanged from Day 0 to Day 7. The ECD was significantly higher in the LPA‐treated group than in the control group on Day 5 and Day 7 (D5 of 4572.2 ± 179.8 vs 3616.7 ± 354.7, *P* < .05; D7 of 4950.0 ± 304.1 vs 3200.0 ± 259.8, *P* < .01). Immunostaining for zonula occludens‐1 (ZO‐1, a CEC marker) revealed that tissue culture did not alter the normal morphology of CECs (Figure 2B). Furthermore, cell proliferation analysed by bromodeoxyuridine (BrdU) labelling was shown to be significantly higher (35.2% ± 16.1% vs 2.8% ± 1.6%, *P* < .01) in the LPA‐treated group than in the control group (Figure 2C).

We established a rabbit model of penetrating keratoplasty (PK) for the functional evaluation of the endothelium in tissue‐cultured corneas. Corneal buttons from 5‐day cultivated donor corneas were used for transplantation into rabbits to replace the central corneal buttons. At 2 weeks after transplantation, no significant differences were observed between donor corneas with and without LPA treatment and mock controls with respect to corneal transparency, protein expression of keratocan (a CSC marker), CEC hexagonal morphology or corneal ECD (mock: 3207.2 ± 128.3; control: 3154.1 ± 237.3; LPA: 3248.7 ± 237.9) (Figure S2). Therefore, we further investigated the healing effect of LPA on wounded CECs. The margin of the CECs and the damage zone were observed using Alizarin Red S staining (Figure 2D). After 2 days of tissue culture, the damage zone had been repopulated by neighbouring CECs. The corneal ECD in the inner (I) and outer (O) damage zones and the cell shape (including aspect ratio and circularity) in the inner damage zone were further examined (Figure 2E). The ECD and cell circularity in the inner damage zone in the LPA‐treated group were significantly higher than those in the control group, while the aspect ratio was significantly lower in the LPA‐treated group than that in the control group (ECD of 1358.3 ± 80.4 vs 900.0 ± 198.4, *P* < .01, circularity of 0.68 ± 0.15 vs 0.43 ± 0.14, *P* < .01 and aspect ratio of 2.39 ± 1.14 vs 4.91 ± 2.43, *P* < .01). Tissue‐cultured damaged corneas were also transplanted into rabbit eyes and observed on Days 0 and 6. Because the damage zone was indistinguishable, the corneal ECD (average of the high‐ and low‐density damage zones) and cell shape in the low‐density zone were examined. In contrast to the consistent corneal opacity observed in the control group, the non‐damaged (MOCK) and LPA‐treated corneas remained transparent. The cell shape in the MOCK group was similar to that in the normal cornea (ECD, 3683.3 ± 187.6; circularity, 0.90 ± 0.03; aspect ratio, 1.22 ± 0.12). In the LPA‐treated group, the ECD and cell circularity in the low‐density zone were significantly higher, while the aspect ratio was significantly lower than those of the control group (ECD of 2200.0 ± 90.1 vs 1700.0 ± 288.3, *P* < .05, circularity of 0.84 ± 0.05 vs 0.69 ± 0.12, *P* < .01 and aspect ratio of 1.35 ± 0.02 vs 2.12 ± 1.13, *P* < .01) (Figure 2F).

### 3.3 Corneal stromal cell‐derived cytokines are involved in LPA‐induced CEC proliferation

**Figure 3:**
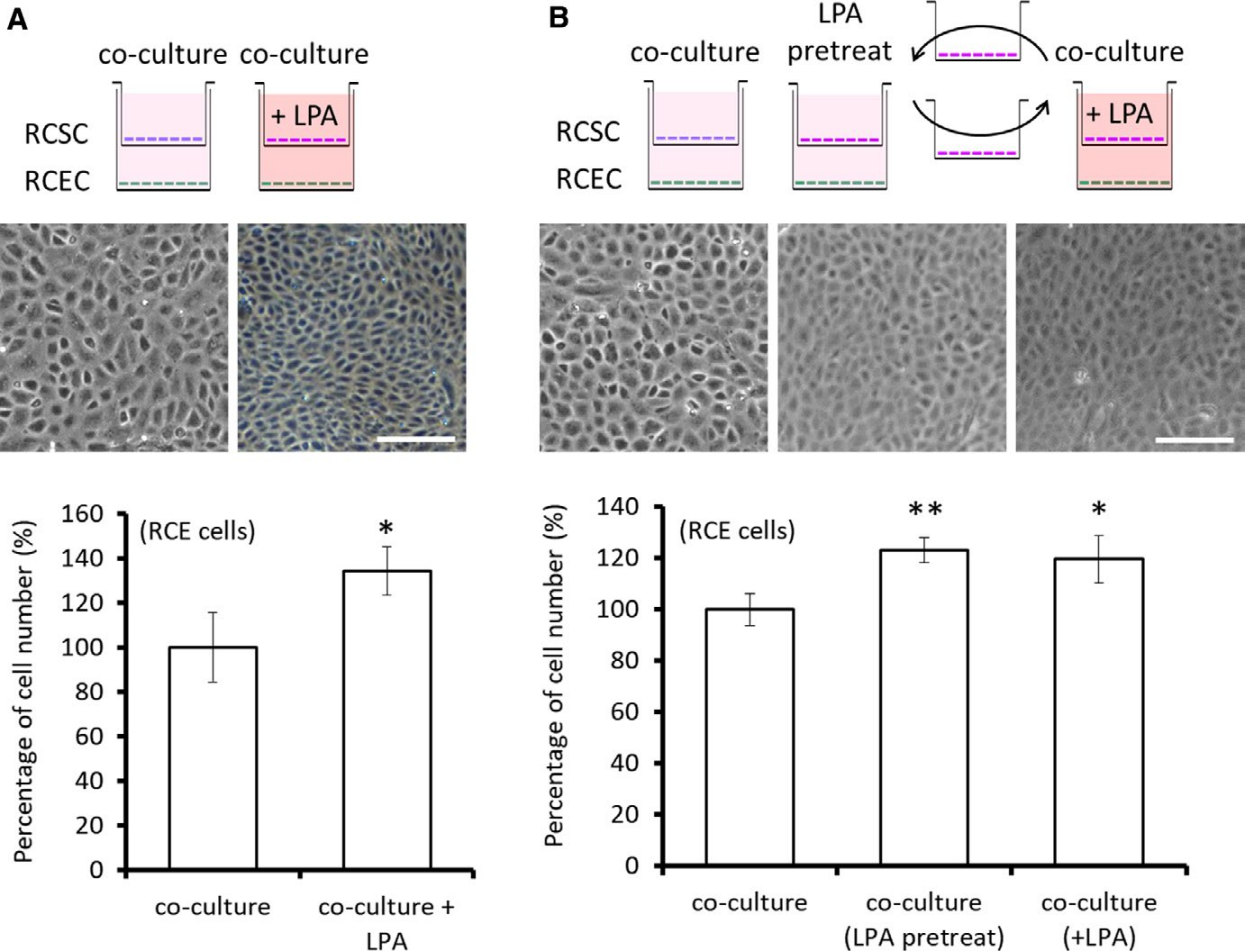
Stromal cell‐mediated regulation involved in LPA‐induced CEC proliferation. A, LPA‐induced proliferation of rabbit corneal endothelial cells (RCECs) was observed in a co‐culture system of RCEC and rabbit corneal stromal cells (RCSCs). Mitomycin C‐pretreated RCSCs plated on transwell inserts were co‐cultured with RCECs and maintained in TC medium with or without LPA (20 μmol/L). After 5 d, the number of RCECs was significantly higher in the LPA‐treated group. B, To investigate whether RCSCs are involved in LPA‐induced RCECs proliferation, the transwell inserts seeding with RCSCs were co‐cultured with RCECs in TC medium; the inserts in LPA‐pretreated group were washed and transposition with the insets in LPA group 3 times per day. After 5 d, the number of RCECs in the LPA‐pretreated and LPA groups was significantly higher than in those not exposed to LPA. The corneal endothelium morphology was observed. Scale bars represent 200 μm. (n = 3; *P < .05, **P < .01)

In the corneal tissue culture environment, LPA added into the medium not only exerted direct effects on CECs but also had inevitable contacts with CSCs. Therefore, we co‐cultivated rabbit CECs (RCECs) and rabbit CSCs (RCSCs) to examine the possible involvement of nearby CSCs on CEC proliferation. In the RCEC‐RCSC co‐cultivation, the number of RCECs was significantly higher in the LPA‐treated group than in the control group (134.3% ± 10.9%, *P* < .05, Figure 3A). To further investigate the role of RCSCs participate in LPA‐induced RCECs proliferation, RCSCs were pretreated with LPA‐containing TC medium and then co‐cultivated with RCECs in TC medium without LPA. The number of RCECs was significantly higher in the LPA‐pretreated group than control group (123.2% ± 4.9%, *P* < .01, Figure 3B).

Because direct cellular contact between CECs and CSCs did not occur in the corneal tissue or in co‐cultivation, we speculated that LPA may modulate secretion from CSCs, thereby affecting the proliferation of CECs. To investigates this hypothesis, we used a cDNA microarray chip and the Cytokine Array G5 chip to determine the secretory‐related factor profile of human CSCs following treatment with LPA (20 μmol/L). In response to LPA treatment, 45 genes in the microarray underwent >10‐fold change in gene expression, including 16 secretory‐related genes (screened by Database for Annotation, Visualization and Integrated Discovery [DAVID]) (Tables S1 and S2). We identified and quantified cytokines expression in the conditioned medium (CM) using the Cytokine Array G5 chip. Of the 80 cytokine targets included on the chip, 4 had significantly higher expression and 25 had significantly lower expression with than without LPA (Table S3). Comparison of these assays is shown in Table 1. CCL20 and IL‐1β have higher secretion after LPA treatment.

**Table 1:** LPA mediates corneal stromal cell‐derived expression and secretion of cytokines (fold change)

Gene symbol	Description	Relative gene expression in stromal cells^a^	Cytokine content in medium^b^
CCL20	Chemokine (C‐C motif) ligand 20	625.99	3.26
IL‐1β	Interleukin 1β	572.05	1.73
CSF3	Colony stimulating factor 3	85.04	0.85
IL‐1α	Interleukin 1α	58.49	0.80

^a^ Gene expression in stromal cells was examined using cDNA microarray. Cytokine genes were screened using DAVID (only overexpressed cytokine genes with a fold change > 50 in the LPA‐treated groups are listed).

^b^ Cytokine content in medium was analysed using a cytokine array. Data are presented as the fold change compared to the control group.

**Figure 4:**
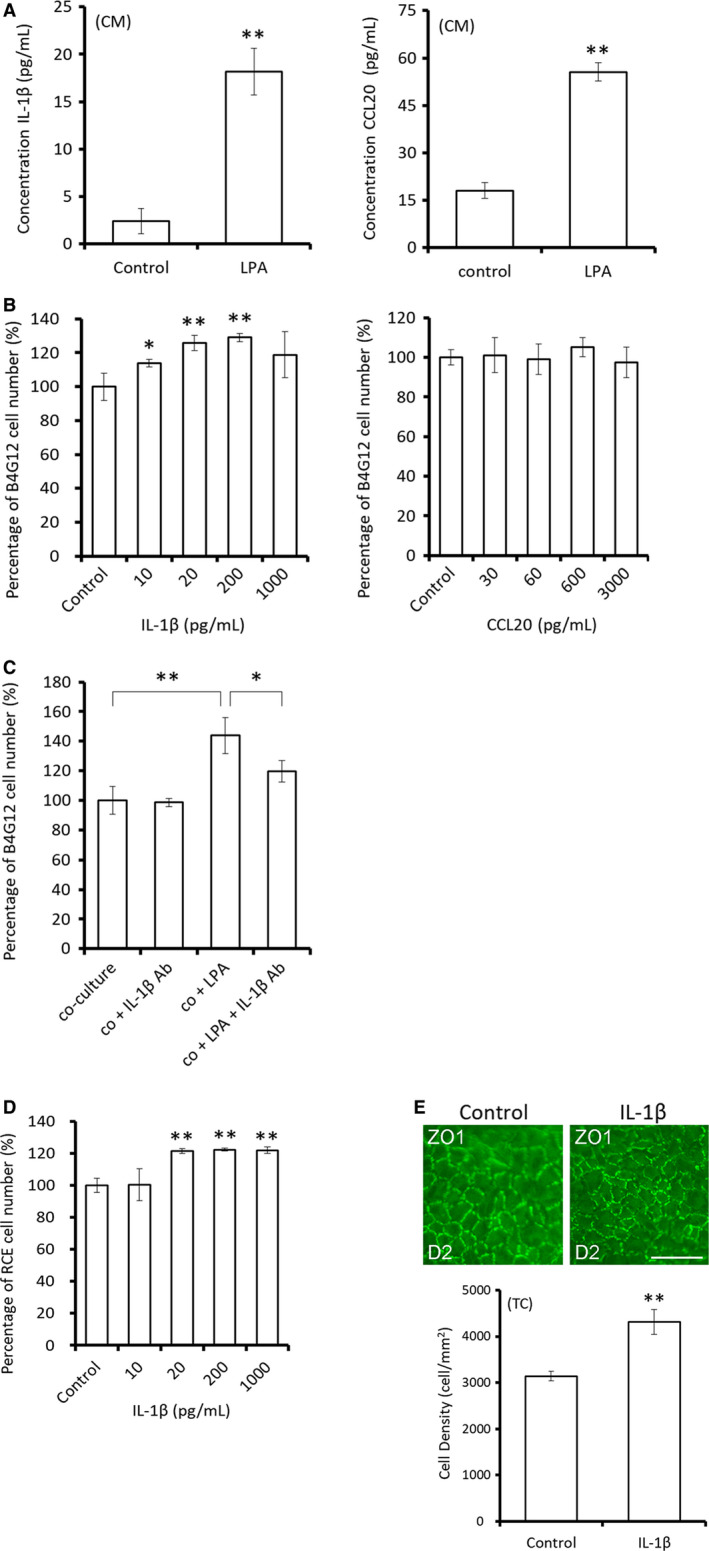
Corneal stromal cell‐derived IL‐1β and CCL20 are involved in LPA‐induced proliferation of corneal endothelial cells (CECs). A, Mitomycin C‐pretreated human corneal stromal cells (HCSCs) were maintained in TC medium with or without 20 μmol/L LPA for 2 d before CM collection. ELISA showed that the concentrations of secreted IL‐1β and CCL20 were both significantly greater in the LPA‐treated group. B, The dose‐dependence of IL‐1β‐ and CCL20‐induced B4G12 cell proliferation was determined by cell counts. The number of cells was significantly higher after treatment of recombinant IL‐1β alone for 2 d. In contrast, no difference was observed in the CCL20‐treated group. C, The involvement of IL‐1β in LPA‐induced proliferation of CECs was investigated via neutralization assay of the CM. Immortalized HCECs (B4G12) co‐cultured with mitomycin C‐pretreated HCSCs in TC medium for 5 d were greater in cell number in the LPA‐treated group (20 μmol/L). Subsequent IL‐1β neutralization with 2 ng/mL IL‐1β‐specific antibody (IL‐1β Ab, clone AS10) significantly attenuated the LPA‐induced proliferation in co‐cultures. D, The proliferation‐promoting effect of IL‐1β on RCECs was also examined 2 d later. The fraction of RCECs in the co‐culture increased significantly with increasing concentrations of IL‐1β (20, 200 or 1000 pg/mL). E, The morphology and ECD of the 200 pg/mL IL‐1β‐treated tissue‐cultured rabbit corneal endothelium was examined via immunostaining for ZO‐1 on Day 2. The corneal ECD was significantly higher in the LPA‐treated cells than that in controls and had the normal hexagonal phenotype typical of RCECs. (n = 3; *P < .5, **P < .05)

The secretion of CCL20 and IL‐1β in the CM was further quantified using enzyme‐linked immunosorbent assay (ELISA). We observed that exposure to 20 μmol/L LPA for 2 days increased the secretion of CCL20 (17.99 ± 2.50 vs 55.53 ± 2.88 pg/mL, *P* < .01) and IL‐1β (2.39 ± 1.35 vs 18.17 ± 2.47 pg/mL, *P* < .01) from human CSCs (Figure 4A). Dose‐dependent recombinant IL‐1β stimulation of elevated cell yield in immortalized HCEC (B4G12) was observed, with the percentage of cells increasing significantly with 10 pg/mL IL‐1β (approximately half the concentration in the medium of the LPA group) and reaching maximum levels at 200 pg/mL (Figure 4B). In contrast, no significant difference was observed in the CCL20 treatment group.

To confirm the involvement of IL‐1β in LPA‐induced CEC proliferation, 2 ng/mL of IL‐1β antibody was used for the neutralization assay. B4G12 cells were co‐cultivated with HCSCs in TC medium for 2 days, resulting in a significant increase in B4G12 cell number in the LPA (20 μmol/L) group compared with that in the control group (100.0% ± 9.6% vs 143.9% ± 12.2%, *P* < .01). While IL‐1β Ab had no effect on overall cell yield in the non‐LPA‐treated co‐culture, IL‐1β neutralization significantly attenuated LPA‐induced elevated cell yield in co‐culture (143.9% ± 12.2% vs 119.7% ± 7.3%, *P* < .05) (Figure 4C). The effect of recombinant IL‐1β on the cell yield of RCECs was also examined. Despite the lack of a dose‐response effect, the percentage of cells relative to controls significantly increased 2 days after the addition of IL‐1β at 20, 200, or 1000 ng/mL (Figure 4D). To confirm the efficacy of IL‐1β treatment in the airlifted tissue culture system, 200 ng/mL IL‐1β was added to the TC medium for 2 days. The rabbit ECD increased significantly (3141.7 ± 104.1 vs 4316.7 ± 267.3, *P* < .01), and immunostaining for ZO‐1 indicated that the normal phenotype was preserved (Figure 4E).

### 3.4 LPA and IL‐1β induced human CEC proliferation in tissue culture

**Figure 5:**
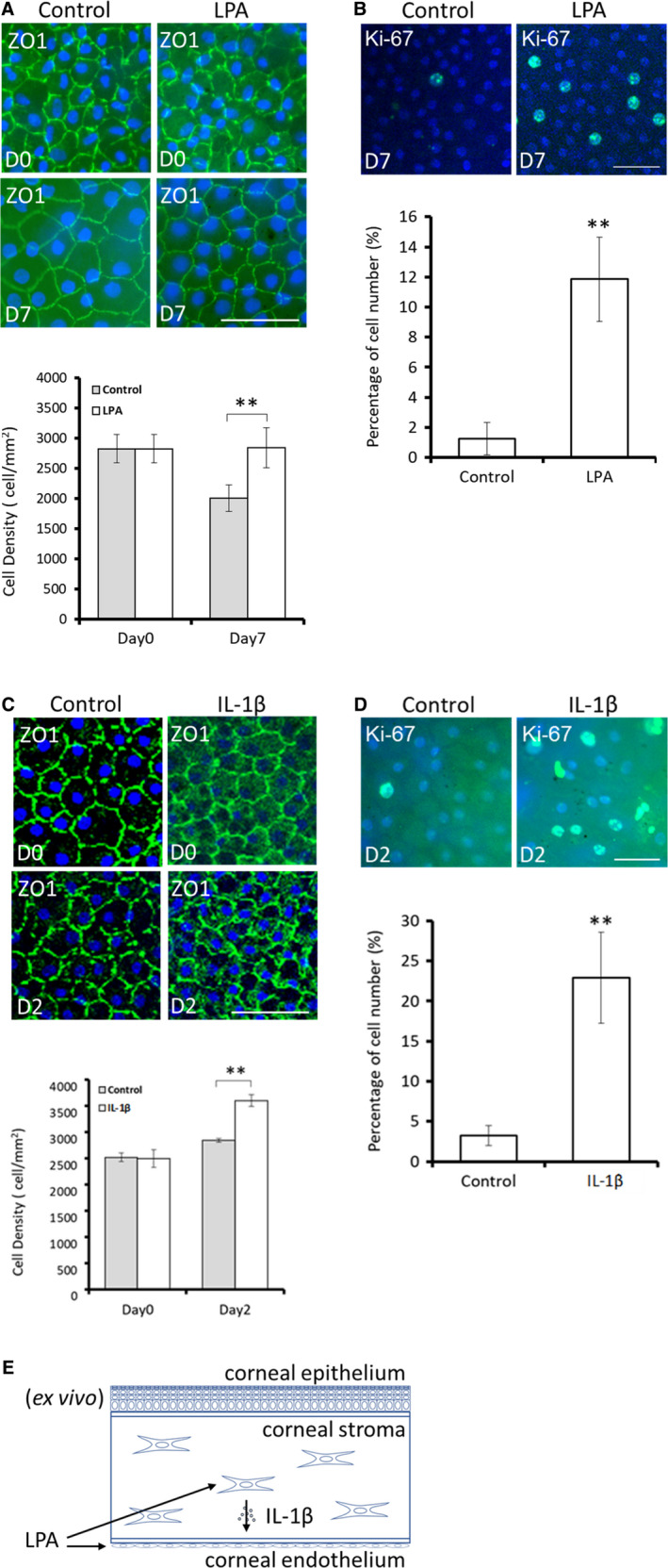
LPA and IL‐1β increase human corneal endothelial cell (HCEC) proliferation in tissue culture. A‐B, Human donor tissues were placed endothelial side up in tissue culture medium with or without LPA (20 μmol/L). The morphology and cell density of tissue‐cultured corneal endothelium were observed via immunostaining for ZO‐1 on Days 0 and 7, indicating a normal hexagonal phenotype. The corneal endothelial cell density was significantly greater in the LPA‐treated group than in the control group on Day 7. Cell proliferation was examined via immunostaining for Ki‐67 on Day 7, demonstrating more Ki‐67‐positive cells in LPA‐treated tissue than in controls. C‐D, IL‐1β‐treated human corneal tissues (200 pg/mL, 2 days) exhibited a normal hexagonal phenotype. The corneal endothelial cell density (ECD) and number of Ki‐67‐positive cells were significantly higher in the IL‐1β‐treated tissues than in controls. Nuclei were counterstained with Hoechst 33342. Scale bars represent 50 μm. (n = 3; **P < .05). E, Schematic diagram showing an indirect effect of LPA on CEC proliferation mediated by the stimulation of IL‐1β secretion from stromal cells

We also observed the effects of LPA on human corneal tissues in ex vivo culture. In contrast to the similarity in ECD between treatments on Day 0, the corneal ECD was significantly lower in the control group than in the LPA‐treated group on Day 7 (2004.7 ± 220.2 vs 2848.8 ± 331.7, *P* < .01) (Figure 5A). Immunostaining for ZO‐1, a CEC marker, revealed that the morphology of CECs was not altered in tissue culture. Additionally, more Ki‐67‐positive cells were observed in the LPA‐treated group than in the control group (11.86% ± 2.80% vs 1.25% ± 1.09%, *P* < .01), indicating that cell proliferation increased after LPA treatment (Figure 5B). Human corneal tissues treated with IL‐1β (200 pg/mL) for 2 days were also examined (Figure 5C,D). HCECs exhibited a normal hexagonal phenotype. The corneal ECD and the number of Ki‐67‐positive cells were significantly higher in the IL‐1β‐treated group than in the control group. (ECD: 3600 ± 108.97 vs 2842 ± 38.19, *P* < .01; Ki‐67:22.89% ± 5.68% vs 3.25% ± 1.24%, *P* < .01).

In summary, we established a tissue culture model in which the corneal ECD was significantly increased after the addition of LPA. We also observed an indirect effect via stimulation of IL‐1β secretion from stromal cells (Figure 5E).

## 4 DISCUSSION

In this study, we established a corneal tissue culture system and observed that the addition of LPA to the TC medium increased the corneal ECD, CEC proliferation and wound healing. We also observed an indirect effect of LPA on the proliferation of CECs via stimulation of IL‐1β secretion from stromal cells in the tissue culture environment (Figure 4F). This novel protocol of tissue culture may be applicable for eye banks to optimize corneal grafting and contribute in regenerative medicine.

To facilitate the rapid expansion of in vitro‐cultivated HCECs, we previously formulated a growth factor‐supplemented HCEC medium for in vitro culture.^12^ However, ex vivo‐cultivated corneal tissues sloughed off the substrate and became apoptotic when grown in this HCEC medium. In contrast, these cells remained attached in TC medium (Figure 1A). We hypothesize that CSCs in the tissue serve as feeder cells, providing the necessary supplements for growth in regular TC medium. In contrast, during ex vivo cultivation of corneal tissues in the HCEC medium, the more complex formulation of ingredients likely induced an increase in the production of active metabolites. With more frequent replacement of the culture medium, cell survival increased (Figure 1A), likely resulting from prevention of the Warburg effect, in which metabolic stress leads to cellular apoptosis.^34^

Thieme et al^26^ used platelet lysate to induce the proliferation of HCECs, but could only reduce ECD loss in the immersed tissue culture model. In this study, enhanced CEC proliferation and ECD were detected in the LPA‐treated rabbit corneas of the airlift tissue culture (Figure 2), while only increased Ki‐67 expression was detected in the LPA‐treated human corneal tissues of immersed tissue culture (Figure 5). This discrepancy in the findings could be attributed to the limited use of only human corneoscleral rim specimens, making airlift manipulation impossible and resulting in stromal oedema in immersed culture, thus compromising the results of ECD. Moreover, we aim to develop culture medium without the need for airlift in future studies, which may be useful for future clinical applications.

Small molecules, such as ROCK inhibitor (Y27632),^9^, ^32^, ^33^ p120‐catenin siRNA (si‐p120)^11^ and LPA,^12^ have been reported to promote the proliferation of HCECs in in vitro culture model. However, in ex vivo tissue culture model, elevated rabbit corneal ECD was only observed in the LPA treatment group (Figure S1 and Figure 2). Y27632 showed promising wound healing effects in the context of massive endothelial sloughing, such as occurs in monkey transcorneal freezing, rabbit mechanical corneal scraping and human Fuchs' dystrophy.^35^, ^36^ However, Y27632 failed to promote proliferation in confluent HCECs, although adhesion and wound healing were enhanced.^37^ In Figure S1, ECD elevation was not detected in the intact corneal endothelium following treatment with Y27632, probably because the RCECs were confluent. Although si‐p120 was reported to promote proliferation in confluent HCECs in vitro via the RhoA‐ROCK‐non‐canonical BMP‐NFκB pathway,^38^ this molecule could not enhance corneal ECD in our tissue culture system, even when immunostaining for si‐p120 was examined to confirm the transfection efficiency in tissue culture. We propose that, in the tissue culture environment, some crosstalk‐directed compensation for si‐p120 was trigged by secretory factors from the stroma.

Consistent with the observed CSC‐derived cytokine‐mediated epithelial wound healing (epithelial‐stromal interactions),^39^ we observed altered cytokine secretory profiles in the LPA‐treated tissue cultures. Although the functional effect of only IL‐1β was confirmed (Figure 4), the regulatory involvement of other cytokines cannot be ruled out. The modulation of CECs by the corneal stroma cell secretome (endothelial‐stromal interactions) is currently under investigation.

One major safety concern related to this model is whether cell transformation occurs when stromal cells are stimulated. LPA was shown previously to induce actin filament assembly in myofibroblasts but not to alter the phenotype of cultured keratocytes.^40^ In the current study of LPA treatment, keratocan protein expression was not elevated. Although LPA did not cause cell transformation in this study, it was recently reported to promote lung carcinogenesis.^41^ Accordingly, for future clinical applications, LPA should be added only during the tissue culture process, and the donor tissue should be assayed for any remaining LPA before corneal transplantation.

## CONFLICTS OF INTEREST

The authors of this manuscript have no conflicts of interest to disclose.

## AUTHOR CONTRIBUTIONS

H.‐CC, W.‐CW and Y.‐JH contributed to the experimental design; C.‐CH, T.‐TL and Y.‐JJM performed the experiments and statistical analyses; C.‐MC and J.‐YL provided technical and material support; H.‐C.C and Y.‐JH contributed to writing and reviewing the manuscript; H.‐CC and W.‐CW supervised the study. All authors read and approved the manuscript.

## Supporting information

Supplementary Material

## Data Availability

The data that support the findings of this study are available from the corresponding author upon reasonable request.
