# Supplementary material for: Lysophosphatidic acid improves corneal endothelial density in tissue culture by stimulating stromal secretion of interleukin‐1β
Source: J Cell Mol Med. 2020 Apr 24;24(12):6596–608. doi: 10.1111/jcmm.15307 (PMC7299697; doi:10.1111/jcmm.15307)
Supplement: Supplementary file 1 — Supplementary Material [file JCMM-24-6596-s001.docx]

# Supporting Information

# Figure S1. Endothelial cell density (ECD) and cell morphology of the rabbit corneal endothelium in airlift tissue culture under different proliferation-stimulating agents.

Inhibition of rho-associated protein kinase (ROCK) or silencing of p120-catenin has been reported to stimulate *in vitro*-cultivated human CEC proliferation. To investigate these proliferation-stimulating factors in *ex vivo* culture, rabbit corneal tissues were cultured in an airlift tissue culture system. For ROCK inhibitor treatment, Y27632 (Sigma-Aldrich) was used at a concentration of 2 μmole/L. For silencing of p120-catenin, corneal tissues were preincubated with Opti-MEM overnight and subsequently transfected with 100 nmole/L rabbit p120-siRNA (si-p120, 5’CAGAGGTGATCGCCATGCTTGGATT3’) using HiPerFect transfection reagent (Qiagen, Hilden, Germany). Nontargeting siRNA (si-control) was used as a control. After transfection or treatment, the medium was changed every 2 days. Phase contrast micrographs were obtained from Day 0 to Day 7. Corneal ECD was counted on Day 7, which was significantly lower in the Y27632-treated group than in the control group (scale bar represents 50 μm; *n* = 3; ***p* < 0.01).


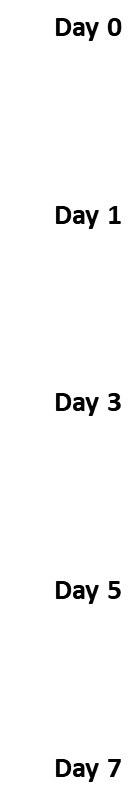

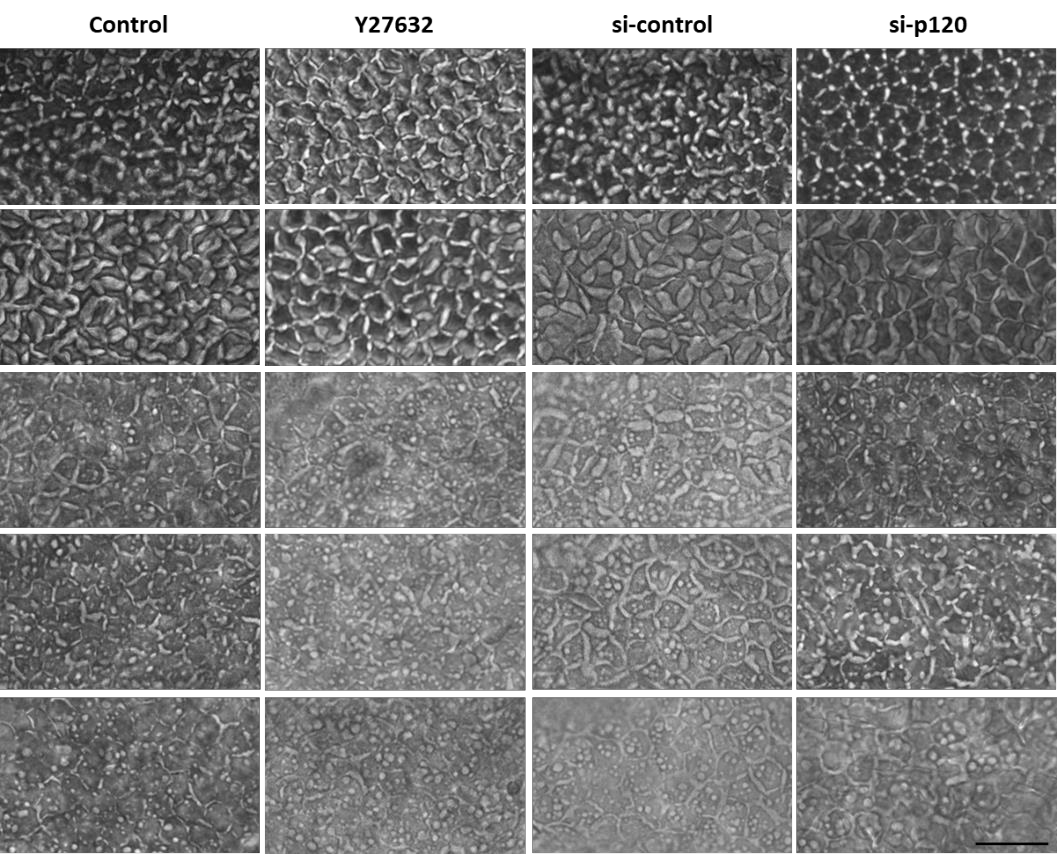


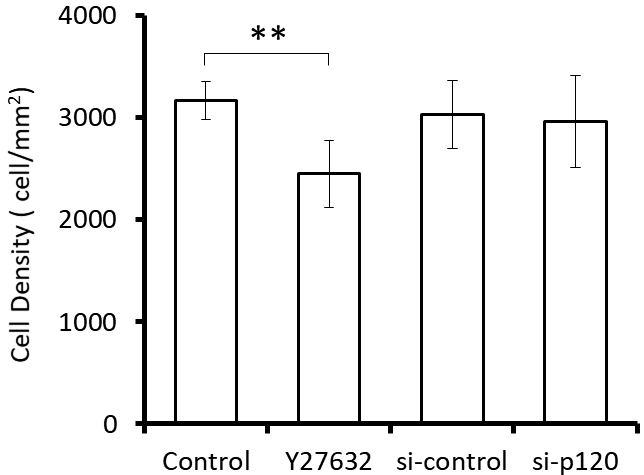


# Figure S2. Functional validation of tissue-cultured corneas using a rabbit penetrating-keratoplasty (PK) model

To verify the endothelial function of tissue-cultured corneas, rabbit corneas were cultured in an

airlift tissue culture system (medium with or without 20 μmol/L LPA) for 5 days, followed by transplantation to rabbits, replacing the central corneal buttons.

(A). External eye photographs were taken on Day 0 and Day 14 to show the clarity of the transplanted corneas. The corneal tissues were then immunostained for ZO-1 (green), indicating that the hexagonal morphology of the corneal endothelial cells was maintained. Corneal ECD was also counted, revealing no significant differences between groups. The scale bar represents 100 μm.

(B). The corneal stroma was further immunoblotted with keratocan (a keratocyte marker). Keratocan expression did not differ significantly between groups, indicating that the phenotype of keratocytes was maintained after tissue culture.

(A). (B).


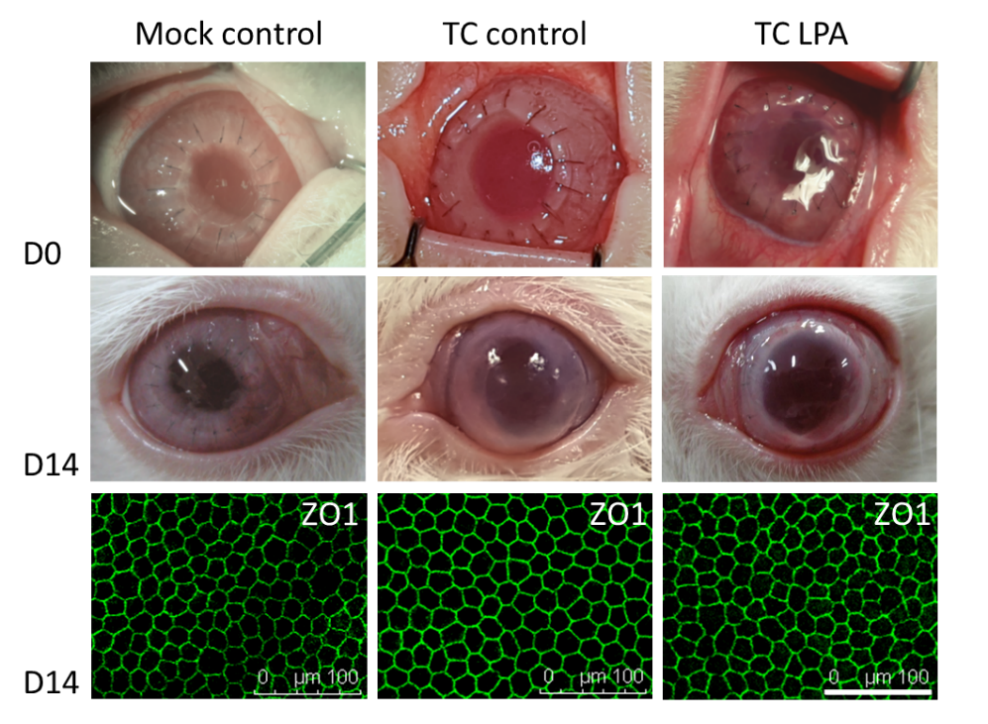


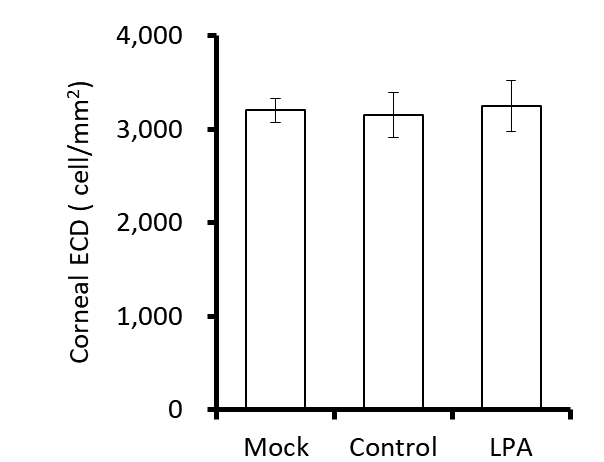

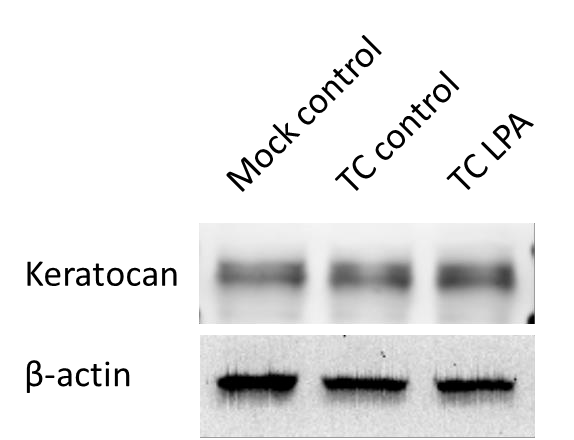


**Table S1.** LPA mediates secreted gene expression in corneal stromal cells

| (Fold change) | >2 | >10 | <2 | <10 |
| --- | --- | --- | --- | --- |
| Total genes | 804 | 45 | 744 | 2 |
| Secreted genes^*^ | 97 | 16 | 64 | 1 |

*Gene expression in LPA-treated stromal cells was examined by cDNA microarray. The secreted genes were screened by the Database for Annotation, Visualization and Integrated Discovery (DAVID).

**Table S2.** Overexpressed secreted genes with a fold change > 10 in the LPA-treated groups

| Gene symbol | Description | Relative gene expression  in stromal cells |
| --- | --- | --- |
| CCL20 | Chemokine (C-C motif) ligand 20 | 625.99 |
| IL1B | Interleukin 1 beta | 572.05 |
| CSF3 | Colony stimulating factor 3 | 85.04 |
| IL1A | Interleukin 1 alpha | 58.49 |
| OLR1 | Oxidized low density lipoprotein receptor 1 | 24.25 |
| CXCL5 | Chemokine (C-X-C motif) ligand 5 | 22.32 |
| CXCL10 | Chemokine (C-X-C motif) ligand 10 | 20.97 |
| INHBA | Inhibin beta A | 20.97 |
| ESM1 | Endothelial cell-specific molecule 1 | 20.11 |
| CCL5 | Chemokine (C-C motif) ligand 5 | 17.63 |
| IL1RN | Interleukin 1 receptor antagonist | 17.39 |
| CCL8 | Chemokine (C-C motif) ligand 8 | 15.89 |
| IL7R | Interleukin 7 receptor | 15.03 |
| CCL7 | Chemokine (C-C motif) ligand 7 | 11.39 |
| CXCL3 | Chemokine (C-X-C motif) ligand 3 | 11.31 |
| EPGN | Epithelial mitogen | 10.85 |

**Table S3.** LPA mediates corneal stromal-cell–derived secretion of cytokines

| **(Up-regulated)** | | | | | | | |
| --- | --- | --- | --- | --- | --- | --- | --- |
| CCL20 (3.27) | GCP-2 (1.74) | IL-1 b (1.73) | TIMP-1 (1.52) |  |  |  |  |
| **(Unchanged)** | | | | | | | |
| LIGHT (1.20) | Eotaxin (1.14) | SDF-1 (1.12) | TIMP-2 (1.06) | OPG (1.04) | EGF (1.03) | CXCL10 (1.01) | HGF (0.99) |
| ANG (0.97) | VEGF (0.93) | TGF-b2 (0.92) | MIG (0.89) | TNF-b (0.89) | NT-3 (0.88) | TNF-a (0.88) | IL-2 (0.87) |
| IL-8 (0.87) | LIF (0.87) | MCP-1 (0.87) | MIF (0.87) | IL-7 (0.86) | CSF3 (0.85) | IL-12 (0.85) | IL-15 (0.84) |
| CCL7 (0.84) | CXCL3 (0.83) | FLT3LG (0.82) | GM-CSF (0.82) | IFN-g (0.82) | IL-10 (0.82) | IL-13 (0.81) | IL-1a (0.80) |
| IL-5 (0.80) | IL-3 (0.78) | IL-6 (0.78) | TGF-b1 (0.78) | IGF-I (0.75) | IGFBP-4 (0.75) | Leptin (0.73) | SCF (0.73) |
| GRO-a (0.71) | IGFBP-2 (0.69) | OSM (0.69) | PARC (0.69) | PIGF (0.69) | THPO (0.69) | TARC (0.67) |  |
| **(Down-regulated)** | | | | | | | |
| BDNF (0.60) | IL-4 (0.58) | Eotaxin-2 (0.59) | NAP-2 (0.59) | NT-4 (0.59) | Eotaxin-3 (0.57) | CCL8 (0.54) | GDNF (0.52) |
| MCP-4 (0.52) | MDC (0.50) | BLC (0.49) | FGF-9 (0.47) | MCSF (0.47) | SPP1 (0.46) | Ck b 8-1 (0.45) | CXCL5 (0.45) |
| FGF-7 (0.45) | PDGF-BB (0.45) | IGFBP-1 (0.41) | IGFBP-3 (0.39) | IL-16 (0.34) | I-309 (0.25) | CCL5 (0.23) | CX3CL1 (0.22) |
| TGF-b3 (0.15) | FGF-4 (0.13) | FGF-6 (0.01) | MIP-1b (0.01) | MIP-1d (0.01) |  |  |  |

Cytokine contents in medium were analyzed using the Human Cytokine Array G5 [AAH-CYT-G5]. Data are expressed as fold changes compared to control group.
